# Therapeutic Actions of the Thiazolidinediones in Alzheimer's Disease

**DOI:** 10.1155/2015/957248

**Published:** 2015-10-26

**Authors:** María José Pérez, Rodrigo A. Quintanilla

**Affiliations:** ^1^Laboratory of Neurodegenerative Diseases, Centro de Investigación Biomédica, Universidad Autónoma de Chile, San Miguel, 8900000 Santiago, Chile; ^2^Dirección de Investigación, Universidad Científica del Sur, 15074 Lima, Peru

## Abstract

Alzheimer's disease (AD) is a multifactorial metabolic brain disorder characterized by protein aggregates, synaptic failure, and cognitive impairment. In the AD brain is common to observe the accumulation of senile plaques formed by amyloid-beta (A*β*) peptide and the neurofibrillary tangles composed of modified tau protein, which both lead to cellular damage and progressive neurodegeneration. Currently, there is no effective therapy for AD; however several studies have shown that the treatments with the peroxisome proliferators activated receptor-gamma (PPAR*γ*) agonists known as thiazolidinedione drugs (TZDs), like rosiglitazone and pioglitazone, attenuate neurodegeneration and improve cognition in mouse models and patients with mild-to-moderate AD. Furthermore, studies on animal models have shown that TZDs inhibit neuroinflammation, facilitate amyloid-*β* plaque clearance, enhance mitochondrial function, improve synaptic plasticity, and, more recently, attenuate tau hyperphosphorylation. How TZDs may improve or reduce these pathologic signs of AD and what the mechanisms and the implicated pathways in which these drugs work are are questions that remain to be answered. However, in this review, we will discuss several cellular targets, in which TZDs can be acting against the neurodegeneration.

## 1. Introduction

AD is one of the most common neurodegenerative diseases that affect elderly population worldwide [1]. Clinically, AD is recognized as a progressive and neurodegenerative disorder that causes deterioration of memory, judgment, and reasoning in patients [2]. In addition, this disease is a complex disorder caused by copathogenic interactions between various constituents such as genetic, epigenetic, and environmental factors [3]. From a genetic point of view, AD may be classified into two types, familial cases (FAD) with autosomal dominant inheritance and an early onset that represent nearly about 4–8% of the AD cases and sporadic Alzheimer's disease (SAD) with less apparent or without familial cause and usually is present at later onset age [4]. Although the sporadic AD is the most common form of the disease, the pathogenesis is not well understood.

Neuropathological features of AD are the accumulation of misfolded proteins in the aging brain [2, 3]. These aggregates are formed amyloid-beta (A*β*) and phosphorylated tau protein in the form of intraneuronal neurofibrillary tangles (NFTs) [2, 3]. These misfolded proteins aggregates lead to oxidative and inflammatory damage, which in turn may result in energy failure and synaptic dysfunction [2, 5]. However, several recent studies suggest that AD could be a degenerative metabolic disease, caused by relevant physiological alterations like diabetes, hypercholesterolemia, and metabolic syndrome (MS) [6, 7]. For example, studies made on patients and preclinical studies showed a significant impairment in brain insulin responsiveness, glucose utilization, and energy metabolism [8, 9]. Also, important studies established a link between the unhealthy nutritional behavior and AD [10]. These disorders correspond to metabolic diseases such as obesity, hypertension, and diabetes mellitus type 2 (T2DM) [10].

T2DM is one of the most prevalent chronic diseases related to MS and is characterized by chronic high glucose contained in the blood (hyperglycemia) caused by the inability of the body to produce insulin or the inability of the cells to respond to the insulin produced by the pancreas [11]. Interestingly, T2DM has been indicated as a risk factor for the development of AD, and recent evidence suggests that the choice of drug treatment may influence the risk for patients with T2DM to develop AD [12]. In fact, new findings of Heneka and colleagues suggest that medication with pioglitazone and rosiglitazone (TZDs) is associated with a lower risk of dementia for T2DM patients, when these are compared to patients treated with metformin or insulin [13].

TZDs are PPAR*γ* agonists used as antidiabetic drugs, whose mechanism of action induces a decrease in plasma free fatty acid concentrations and fasting hyperglycemia via an insulin-sensitizing effect [14]. PPARs are nuclear lipid sensors capable of adapting gene expression to integrate various lipid signals coming from intra- and extracellular environment [15]. Also, PPAR*γ* is the most studied isoform of the PPAR family, and it is the one that has showed the most promising neuroprotective effects in various models of neurodegenerative disorders [16].

For instance, in patients with AD, several clinical studies have tested the efficacy of TZDs treatments, indicating that some of these drugs significantly reduce the incidence of dementia in people with diabetes [13]. Recently, meta-analysis studies showed that pioglitazone treatment might be therapeutically beneficial in early stages of AD and for patients with mild-to-moderate AD [17]. Further analysis of these studies showed a significant reduction in amyloid-beta and tau pathology measured in cerebral blood flow samples from AD patients (CBF) [17]. This data, along with several other studies that have shown that TZDs increase memory and learning in several animal models of AD, strongly suggests that TZDs should be considered as a valid target against AD. However, the implicated pathways where the drugs work remain unknown.

In this review, we will focus on the recent progress of how TZDs selectively modulated different cellular targets in AD. We will center our attention on the possible effects and improvement against neuroinflammation, amyloid-*β* clearance, mitochondrial dysfunction, synaptic impairment, and tau pathology.

## 2. Activators of PPAR*γ* against Neurodegeneration in AD

### 2.1. Thiazolidinediones and Cognitive Improvement

One of the major clinical signs in the patients with AD is the cognitive decline that is presented by a group of symptoms that includes memory loss, language difficulties, and executive dysfunction [18]. Also, patients may present noncognitive related impairment, such as psychiatric symptoms and behavioral disturbances, depression, hallucinations, delusion, and agitation [18]. Suitable animal models of neurodegenerative conditions are valuable to understand the pathophysiology of dementia and the development of new therapeutic strategies [19]. Indeed, we will describe several AD mouse models that regularly present cognitive decline but showed a significant improvement in memory and cognitive tasks after the treatment with TZDs.

As we previously described, TZDs are known as insulin sensitizers with beneficial effects against neurodegeneration. However, patients with diabetes under TZDs treatment presented cognitive decline [13]. In contrast, studies on a rat model of type 2 diabetes treated with rosiglitazone showed a significant improvement in spatial learning and memory task [20]. Apparently, this effect is related to the regulation of the insulin signaling pathway, which involved a decrease in the expression of IR, IRS-1, AKB, p-CREB, and Bcl-2 in the hippocampal neurons from the rats [20]. In other studies, animal models treatment with TZDs showed similar results; for example, injection of rosiglitazone directly into the brain of Wistar rats significantly ameliorates the memory impairment induced by the presence of A*β* oligomers [21]. Also, in the A/T bitransgenic mouse that rapidly forms senile plaques and overproduces A*β* and TGF-*β*1, the treatment with pioglitazone improved reversal learning in the adult mice [22].

In complementary studies on the AD transgenic mouse J20 (which overexpress human amyloid precursor with the Swedish and Indiana family AD mutations), the treatment with rosiglitazone reduced memory deficits in the object recognition and the Morris water maze (MWM) tests, similar to the performance of the nontransgenic group [23]. In addition, in experiments on the triple transgenic mouse 3xTg-AD (a mouse line that expresses APP, presenilin 2, and human tau), it was found that pioglitazone treatment improved learning in a hippocampal-dependent task [24] and this was evaluated with the MWM test [25]. Consistent with this, a study on twelve-month-old APP/PS1 mice treated with pioglitazone for nine days resulted in significant behavioral improvement evaluated by a contextual fear-conditioning assay [26]. Also, the pioglitazone treatment reversed noncognitive behavioral deficits and restored distance and speed traveled in an open field [26]. Complementary studies on the same transgenic mouse model showed that treatment with rosiglitazone reduced the cognitive deficit in the reversal phase of the MWM tests [27]. In addition, treatment with pioglitazone improved spatial memory using the same test [28], and this also was seen in the same mouse model treated with rosiglitazone and lithium; both drugs can activate Wnt signaling and inhibit the glycogen synthase kinase 3*β* (GSK-3*β*), a kinase that is a key in the progression of the AD [29]. Despite the studies presented above, the therapeutic mechanism by which PPAR*γ* agonist led to improved cognition remains unknown.

Extracellular signal-regulated protein kinase (ERK) is essential for several forms of hippocampus-dependent learning and memory and that apparently depends on ERK phosphorylation through transcriptional regulation of specific target genes [30]. Several studies suggest that ERK-dependent memory mechanism is impaired in AD [30]. For example, Jahrling and colleagues have shown that treatment of Tg2576 mouse (AD model) with rosiglitazone induced recruitment of PPAR*γ* to pERK during memory consolidation and that complex formation correlated with an improvement in cognitive performance in the AD mouse model [30]. Another mechanism of action for PPAR*γ* activation may be the improvement of the acetylcholine (ACh) reduction, which leads to a cholinergic dysfunction in AD [5]. Evidence, obtained from a mouse model of the cholinergic deficit in the brain, indicated that pioglitazone improved learning and memory retention in the MWM tests and increased the performance in the passive avoidance test [31]. ACh level mediated by the synthesis of ACh regulatory enzymes was increased in the hippocampus and cortex of the dementia mouse model [31], and the same effect occurs in Wistar rats injected with *β*-amyloid and treated with pioglitazone [32].

Several other mechanisms, different from those already described, could be involved in the cognitive enhancement mediated by the therapeutic action of TZDs in various models of Alzheimer's disease. Some of these pathways that seem to have a significant contribution to the amelioration of the symptoms in AD will be described in the next sections.

### 2.2. Thiazolidinediones and Neuroinflammation

Cerebral inflammation occurs through the activation of microglia and recruited peripheral macrophages, and both may promote neurotoxicity and participate in the progression of neurodegeneration [33]. Amyloid-beta aggregates and neurofibrillary tangle can generate an innate immune system response in AD; for this reason, neuroinflammation is considered an important player in the pathogenesis of AD [34]. Interestingly, PPAR*γ* activation has been shown to have a potent anti-inflammatory effect [35]. Thus, several groups had investigated the effects of TZDs treatment against the inflammatory response in AD.

The initial studies reviewed by Heneka and colleagues, which explored the effects mediated by PPAR*γ* on neurological disorders, showed that selective inflammatory drugs, which do not exert any PPAR*γ* ligand activity, failed to show a clinical benefit in AD [36]. Interestingly, the activation of PPAR*γ in vitro* and* in vivo* suppressed proinflammatory response through an antagonism of the transcription factor nuclear factor *κ*B (NF*κ*B) or AP-1 and, with that, prevented the activation of microglia mediated by A*β* [35, 36]. More recently, the effects of TZDs against neuroinflammation in animal and brain cell models have been investigated. For example, the treatment with pioglitazone reduced astrocytes and microglial activation in the cortex and hippocampus of the A/T mouse that overproduces A*β* and TGF-*β*1 [22]. At the same time, injection of rosiglitazone into the brain of Wistar rats, previously treated with A*β* oligomers, prevented the increase of inflammatory cytokines levels and this is related to an improvement in cognitive decline and to prevention of microglia activation [21]. Interestingly, the same effects occur in the AD transgenic mouse models J20 and APP/PS1 with previous oral administration with rosiglitazone [23, 27]. A similar study, in which pioglitazone was administrated by oral way, showed a significant inhibition of the IL-6 and TNF*α* increased levels induced by A*β* stimulation [32]. Additionally, in the Cdk5 conditional knockout mice that showed activation of microglia and astrocytes, pioglitazone treatment resulted in a significant reduction in the activation of microglia and astrocytes and neuronal loss associated with a better survival rate [37]. This is important, because Cdk5 is a protein kinase, whose deregulation contributes to synaptic loss and tau hyperphosphorylation in the AT8 epitope (present in the AD brain) after stimulation of A*β* fibrils [38, 39]. All these studies indicate that inhibition of inflammatory response is involved in the beneficial roles of TZDs treatment in AD, and this response is mediated by microglia and astrocytes.

Further studies by Mandrekar-Colucci and colleagues evaluated the expression of the microglial activation marker M1 by immunofluorescence [26]. M1 is expressed in activated microglia and changes microglia morphology generating a highly polarized phenotype and that produces inflammatory cytokines and reactive oxygen species (ROS) [40]. These studies showed a significant reduction of the immunofluorescence intensity of microglial activator marker M1 in the surrounding area of amyloid deposits in brain samples of 12-month-old APP/PS1 mice treated with pioglitazone [26]. On the other hand, the expression of M2 marker produces a phenotype with a barely polarized microglia that generates anti-inflammatory cytokines promoting phagocytosis and tissue repair [26, 40]. Using the same mouse model, Mandrekar-Colucci and colleagues showed an elevated expression of M2 markers in 12-month-old APP/PS1 mice treated with pioglitazone and a reduction in the levels of inflammatory cytokines and amyloid plaques [26]. Furthermore, pioglitazone treatment reduced the levels of GFAP-immunoreactive astrocytes that were surrounding amyloid plaques in APP/PS1 mice at 6 and 12 months of age, and internalized A*β* peptides only in astrocytes of pioglitazone-treated animals interestingly were found [26].

Altogether, these data suggest that TZDs treatment not only reduced the inflammatory response by microglia and astrocytes but also facilitated the removal of A*β* deposits presumably through enhancing the phagocytic activity of these cells. However, it is not clear if TZDs protective effects are only produced by PPAR*γ*. The mechanism involved in amyloid clearance will be discussed in the next section.

### 2.3. Thiazolidinediones and Amyloid Clearance

As we previously mentioned, in AD the cerebral plaques that are loaded with deposits of A*β* are an important pathological feature of AD, because their presence can initiate a cascade of events that includes neurodegeneration and the formation of neurofibrillary tangles through the hyperphosphorylation of tau protein [2, 5]. A*β* is a 39–43 amino acid peptide that is proteolytically derived from the amyloid precursor protein (APP) through enzymatic action of *β*-secretase and *γ*-secretase [5]. An imbalance between the production and clearance of A*β* causes protein accumulation and a pathological condition [5]. Interestingly, several independent studies that we will present next have shown that the treatment with TZDs in animal models of AD decreases the A*β* accumulation. For example, Heneka et al. showed that oral treatment of APP transgenic mice with pioglitazone reduces the transcription and expression of BACE1, an enzyme called *β*-secretase that processes APP protein [41]. However, other studies had demonstrated that the amyloidogenic APP processing and A*β* production are not affected by the treatment with rosiglitazone [23] or pioglitazone [26], suggesting that the decrease in A*β* plaque produced by TZDs is related to the amyloid degradation [23, 26].

Apolipoprotein E (ApoE) is a lipoprotein principally expressed in liver and brain and it has been shown to enhance both the degradation and phagocytosis of A*β* in the microglia and astrocytes [5]. In addition, it was demonstrated that A*β* clearance is impaired in AD patients and that the presence of the APOE4 allele is associated with an increased accumulation of A*β*, which is considered a risk factor associated with late onset of FAD [42, 43]. In reference to that, treatment of microglia and astrocytes with pioglitazone increased the intracellular degradation of soluble A*β* in a dose-dependent manner [26]. This can occur because TZDs induce the overexpression of ApoE helping in the amyloid clearance [26]. Also, studies on primary microglial cell cultures showed that pioglitazone induces the activation of PPAR*γ* and the receptor heterodimerization with retinoic X receptor *α* (RXR*α*) regulating the expression of CD36, which induced A*β* clearance mediated for phagocytosis [44]. Complementary studies on N9 mouse microglial cell line showed that the treatments with pioglitazone enhance the phagocytic activity of the cells and reduce the levels of proinflammatory cytokines [45]. Interestingly, a study performed on APP/PS1 mice of 6 and 12 months of age showed that treatment with pioglitazone increased the levels of ATP-binding cassette transporter (ABCA1) and apoE and reduced the soluble and insoluble levels of A*β* by 50% [26]. In addition, the expression and processing levels of APP and of A*β*-degrading enzymes were not affected, suggesting that the changes seen in amyloid deposition were a result of A*β* catabolism [26]. Similar results were obtained with rosiglitazone treatment in J20 mouse model [23] and with the APP/PS1 mice [27]. These studies suggest that TZDs enhance the amyloid clearance in cell lines of microglia and astrocytes treated with A*β* and these effects are related to the activation of the ApoE pathway.

Interestingly, a clinical trial that evaluated the effects of rosiglitazone on more than 500 patients with mild-to-moderate AD resulted in a significant improvement in cognition in patients that did not carry APOE4 allele [46]. In contrast, patients with APOE4 positive treated with rosiglitazone did not show any improvement in standard cognitive test [46]. These observations suggest that the amyloid clearance pathway dependent of TZDs also depends on the expression of functional ApoE [46].

### 2.4. Thiazolidinediones, Mitochondrial Function, and Synaptic Plasticity

Several reports have showed that the exposure to A*β* affects different aspects of mitochondrial function contributing to the AD pathology in the brain [2, 47, 48]. The metabolic disturbances and the oxidative damage are considered the major contributors of mitochondria impairment to the pathogenesis of AD [47, 48]. However, AD is a disorder characterized by synaptic failure, and several studies showed that disturbances in mitochondrial dynamics and function may cause synapse loss in AD [47, 49].

Studies on N2A cells showed that the treatment with rosiglitazone increased the mitochondrial mass and function through the activation of PPAR coactivator 1*α* (PGC1*α*) mediated for the PKA/CREB/AMPK pathway [50]. Additionally, rosiglitazone promoted neurite outgrowth and increased neuronal function [50]. Interestingly, in hippocampal neurons treated with A*β* oligomers, the treatment with rosiglitazone resulted in a prevention of filopodium loss and enhanced synaptic function [50]. Filopodium loss is related to the reduction of dendritic spines and synapses in AD [51]. They also showed that rosiglitazone prevented the inhibition of long-term potentiation (LTP) induced by A*β* oligomers, increasing the density and number of mitochondria in the dendritic spines [51].

In another study, the chronic treatment with pioglitazone attenuated oxidative damage and restored mitochondrial respiratory activity and promotes mitochondrial biogenesis in Wistar rats injected with A*β* [32]. Additionally, in brain extracts from rats treatment with pioglitazone prevented caspase-3 activation and increase of BDNF levels, which is an essential factor for neural cell survival, differentiation, and synaptic activity [32]. Otherwise, in the triple transgenic mouse model of AD (3xTg-AD) it was found that pioglitazone caused a significant increase in the amplitude of the synaptic hyperpolarization, a phenotype typically seen in younger animals, and also they showed a robust potentiation of the excitatory postsynaptic potentials (EPSPs) compared to control animals [24]. These observations suggest that TZDs prevent the impairment of synapse plasticity through the increasing of mitochondria and dendrite spine density. It is worthy to mention that not all the effects observed in mitochondria could be mediated for the direct activation of PPAR*γ*; in fact Colca and colleagues explored the possibility that TZD may act through specific mitochondrial targets and regulate the metabolic environment of the neurons [48].

Numerous studies have showed that Cdk5 is vital in the regulation of synaptic plasticity and induces tau hyperphosphorylation in the AT8 epitope (present in the AD brain) after stimulation of A*β* fibrils [38]. More importantly, current studies showed that PPAR*γ* activation with pioglitazone inhibited Cdk5 activity by decreasing the levels of p35, which is a Cdk5 activator in neurons, in a proteasome-dependent manner [28]. Moreover, blockage of Cdk5 by pioglitazone prevented long-term potentiation (LTP) defects at CA3-CA1 synapses in APP/PS1 mice, which is an important form of synaptic plasticity [28]. These observations are important because lately the use of TZDs showed improvement of mitochondrial function and synapse plasticity and reduction of memory loss. However, interestingly, with the participation of Cdk5, a new possible link opens between their kinase activity and the regulation of tau pathology present in the AD brain.

### 2.5. Thiazolidinediones and Tau Pathology

One of the most important pathological features of AD is the presence of NFTs [1, 2]. These are filamentous inclusions present in hippocampal neurons formed by aggregates of the tau protein in a hyperphosphorylated state [2]. Tau belongs to the family of microtubule-associated protein and plays a role in assembly and stabilization of neuronal microtubules network in a serine/threonine phosphorylation dependent pathway [2]. In fact, a hyperphosphorylation of tau reduces its affinity towards microtubules [5], and this reduction may interfere with axonal transport leading to dysfunction of synapses, neuronal loss, and cognitive impairment [52].

The phosphorylation state of tau results from a balance between kinase-mediated phosphorylations and dephosphorylation by protein phosphatases [2]. Some kinases that participate in this process are as follows: cyclin-dependent kinases (Cdk2 and Cdk5), GSK-3*β*, mitogen-activated protein kinase (MAPK), extracellular signal-regulated protein kinase 1/2 (ERK1/2), c-Jun N-terminal kinase (JNK), Akt, protein kinase a (PKA), and calcium-calmodulin protein kinase 2 (CaMKII). On the other hand, some phosphatases, PP1, PP2A, PP2B, and PP2C, contribute to the dephosphorylation of tau [5].

Regarding the effects of TZDs on tau pathology, there were studies on Chinese hamster ovary (CHO) cells stably transfected with the long isoform of human tau (4Rtau) and they were treated with troglitazone and pioglitazone, in which both treatments showed a reduction in the phosphorylation of Ser202, Ser396, and Ser404 [53]. Furthermore, studies using the neuronal cell line SH-SY5Y transfected with the longest isoform of tau showed a reduction in tau phosphorylation in Ser396 and Ser202 after rosiglitazone treatment but with a reduction of the JNK pathway activity [54]. Another study on the AD transgenic mouse J20 treated with rosiglitazone clearly decreased the number of p-tau aggregates and reduced the tau phosphorylation at the same epitopes [23].

Important studies explored the role of PPAR*γ* activation in tau pathology in a rat model of diabetes 2 (OLETF) [54]. Treatment with rosiglitazone by oral administration reduced the levels of tau phosphorylated at Ser396, Ser199, and Ser202 in the CA3 region of the hippocampus and the phosphorylated form of JNK was also decreased [54]. Interestingly, a reduction in the tau phosphorylation also was seen in diet-induced type 2 diabetes treated with pioglitazone but only in ApoE3 mice, because the ApoE4 animals showed a significant increase in the phosphorylation of tau [55].

Further studies with the 3xTg-AD mouse model showed that pioglitazone significantly decreases tau phosphorylated-positive neurons in the hippocampus and improved their cognitive impairment with the TZD treatment [24]. Also, in a recent study the treatment with pioglitazone or rosiglitazone, in the same mouse model, reduced tau phosphorylation in Ser202, Ser396, Ser404, Ser422, and Thr231 in cerebral cortex and CA1 area of hippocampus [25]. Finally, studies on SH-SY5Y cells and rat primary cortical neurons that were treated with troglitazone, rosiglitazone, and pioglitazone showed a decrease in tau-Thr231 phosphorylation in a dose- and time-dependent manner [56]. They also showed that TZDs inhibited Cdk5 kinase activity by decreasing p35 protein levels in a proteasome-dependent manner and are independent of GSK-3*β*, PKA, and protein phosphatase 2A signaling pathways [56]. Complementary studies showed the same effects on Cdk5 pathway after treatment with pioglitazone in the APP/PS1 mice with a distinct improvement in spatial memory tasks [28]. All these results indicate that PPAR*γ* activation modulates tau pathology, which could be a new target for a therapeutic use of TZDs against the neurodegeneration in AD [28].

## 3. Conclusions

**Figure 1:**
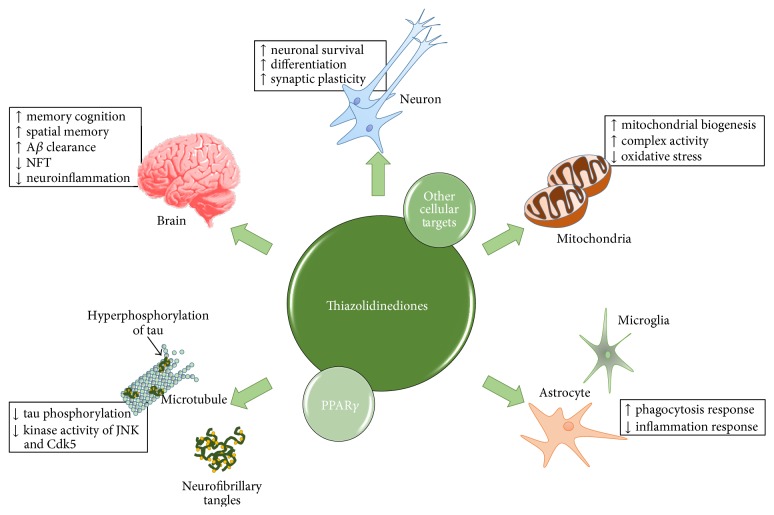
Targets of thiazolidinediones drugs in Alzheimer's disease. The TZDs can bind to PPAR*γ* receptors and other pathways that regulate energy metabolism, in cellular and animal models of AD. In cognition and behavioral test, these drugs increase the memory performance of the animals and also decrease the A*β* deposits accelerating the amyloid plaque clearance. At more cellular levels, TZDs promote the neuronal survival, differentiation, and synaptic plasticity and also increase the phagocytosis and reduce neuroinflammation both in astrocytes and in microglia. In the mitochondria, TZDs induce biogenesis and enhance the mitochondrial function observed by a rise in the respiratory complex activities and reduction of the oxidative stress. Finally, TZDs are capable of reducing tau phosphorylation through the inhibition of different kinases activities and the later formation of the neurofibrillary tangles presented in AD.

The fact that there are still no effective therapies for AD suggests that it is crucial to develop new drugs that deal with the aberrant cellular and molecular signaling pathways affected in this disease. Indeed, the lack of effectiveness of the treatments and inconsistent results indicate that this territory remains unknown [57, 58]. Therefore, a better understanding of the pathogenic events in AD could help to generate a more effective therapy that may interfere early in the development of the disease. Several investigations have revealed the importance of the use of TZDs in the treatment of AD, and some groups had found positive results in clinical trials using some of these drugs [13, 17]. We summarized several mechanisms in which TZDs may be participating, either directly activating PPAR*γ* signaling or regulating alternative cell metabolic pathways (Figure 1). In fact, pioglitazone, rosiglitazone, and troglitazone may act reducing neuroinflammatory damage and A*β* clearance and increasing energy metabolism and enhancing synapse activity and reducing tau pathology. These miscellaneous targets of action could help to understand why these drugs had a positive effect on the cognitive enhancement, not only on animal models of AD but also on a patient with mild-to-moderate AD. Here, we propose that the possibility that one drug may positively act in different targets strongly suggests the use of TZDs against Alzheimer's disease.
